# Long-Term Development of Training Characteristics and Performance-Determining Factors in Elite/International and World-Class Endurance Athletes: A Scoping Review

**DOI:** 10.1007/s40279-023-01850-z

**Published:** 2023-05-13

**Authors:** Hanne C. Staff, Guro Strøm Solli, John O. Osborne, Øyvind Sandbakk

**Affiliations:** 1 School of Sport Sciences, UiT The Arctic University of Norway, Campus Tromsø, Hansine Hansens veg 18, 9019 Tromsø, Norway; 2 Department of Sports Science and Physical Education, Nord University, Bodø, Norway; 3 Department of Neuromedicine and Movement Science, Centre for Elite Sports Research, Norwegian University of Science and Technology, Trondheim, Norway

## Abstract

**Objective:**

In this scoping review, we aimed to 1) identify and evaluate existing research that describes the long-term development of training characteristics and performance-determining factors in male and female endurance athletes reaching an elite/international (Tier 4) or world-class level (Tier 5), 2) summarize the available evidence and 3) point out existing knowledge gaps and provide methodological guidelines for future research in this field.

**Methods:**

This review was conducted following the Joanna Briggs Institute methodology for scoping reviews.

**Results:**

Out of 16772 screened items across a 22-year period (1990-2022), a total of 17 peer-reviewed journal articles met the inclusion criteria and were considered for further analysis. These 17 studies described athletes from seven different sports and seven different countries, with 11 (69%) of the studies being published during the last decade. Of the 109 athletes included in this scoping review, one quarter were women (27%), and three quarters were men (73%). Ten studies included information about the long-term development of training volume and training intensity distribution. A non-linear, year-to-year increase in training volume was found for most athletes, resulting in a subsequent plateau. Furthermore, 11 studies described the development of performance determining factors. Here, most of the studies showed improvements in submaximal variables (e.g., lactate/anaerobic threshold and work economy/efficiency) and maximal performance-indices (e.g., peak speed/watt during performance testing). Conversely, the development of VO2max was inconsistent across studies. No evidence was found regarding possible sex differences in development of training or performance-determining factors among endurance athletes.

**Conclusion:**

Overall, a low number of studies describing the long-term development of training and performance-determining factors is available. This suggests that existing talent development practices in endurance sports are built upon limited scientific evidence. Overall, there is an urgent need for additional long-term studies based on systematic monitoring of athletes from a young age utilizing high-precision, reproducible measurements of training and performance-determining factors.

## Key Points

**Table:** 

Only 17 studies described the long-term development of training characteristics and performance-determining factors of elite/international and world-class athletes, with 16 studies using a retrospective study design, 11 studies being case studies, and the majority of participants being male.
A non-linear year-to-year increase in training volume, mainly driven by increases in low-intensity training until reaching a subsequent plateau at elite/international and world-class level was found for most of the included endurance athletes.
Consistent improvement in maximal performance tests and submaximal performance indices were found for most athletes, while the developments in maximal oxygen uptake were inconsistent across studies.
This scoping review highlights an urgent need for additional long-term studies based on systematic monitoring of athletes and suggests that a common framework is required for comparing results across different long-term studies in endurance sports.

## Introduction

Long-term performance development in endurance sports is determined by a multifaceted interaction of manifold variables. Extensive sport-specific practice, including optimal progression of training volume, frequency, and intensity distribution, is required to stimulate sport-specific adaptive responses. This process normally requires a relatively long period (10–15 years) of dedicated training, although recent studies report considerable variation within and across sports in the amount of training and the time needed to reach elite and super-elite levels [1–4]. In addition to the obvious role of the genetic potential, the realization of athletes’ potential is also influenced by motivation, skillset and experience of the athlete and coach, training peers, supporting staff, training environment and facilities, well-being, and life balance [5, 6].

The training characteristics among elite/international and world-class athletes in endurance sports have been widely described in retrospective studies [7–10]. The outcomes from this research have emphasized the importance of high-endurance training volumes (TV) with sport-specific differences owing to variations in muscular loads and injury risks across exercise modalities [10]. Furthermore, there is an established consensus that a relatively long period of dedicated training is required to tolerate these TV and reach an elite level [4, 11–13]. Accordingly, gradual progression in TV is required to tolerate and respond positively to the overall training load. However, training load can also be manipulated by changing the intensity and/or frequency of training, although limited evidence exists on how the progressive increase in these factors interacts to provide the best possible training stimulus and to avoid setbacks, thereby ensuring continuity to optimize the development of physiological factors and performance [9, 14].

Describing and comparing the intensity distribution of endurance training (TID) across different studies and athletes necessitates a standardized intensity scale. Here, a three-zone model is often used, with the zones referred to as: low-intensity training (LIT), moderate-intensity training (MIT), and high-intensity training (HIT). Although both conceptual and practical challenges are associated with the division of intensity zones, the separation of each zone using reproducible blood lactate anchor points, combined with corresponding heart rate and ratings of perceived exertion, is arguably the most effective available method [9, 15]. Other methods that are used to determine intensity zones include ventilatory thresholds or critical power [16, 17]. Although there are differences in the methods for quantifying training intensity, there seems to be similarities in the basic TID patterns selected by successful endurance athletes [9]. Previous studies report that the training of successful endurance athletes include 70–90% LIT, with the remaining 10–30% performed as MIT and HIT [9, 18, 19]. This variation in TID is likely caused by differences in the demands of the examined sports, individual development areas, and the methodology used to determine LIT, MIT, and HIT [10, 20, 21]. Still, it is unclear if the same TID should be employed in all stages of the development process in an endurance athlete’s career.

Successful endurance performance is characterized by high levels of maximal oxygen uptake (*V*O_2max_), anaerobic threshold or lactate threshold, and work economy or efficiency [22]. However, the long-term development of these performance-determining factors is influenced by various aspects such as training, psychophysiological maturation, and sex, resulting in different developmental patterns throughout an athlete’s career [23]. Therefore, an overview of the studies including information about the long-term development of training characteristics and performance-determining factors of elite/international and world-class athletes would provide a starting point for better understanding the long-term development process of endurance athletes.

Accordingly, this scoping review aimed to (1) identify and evaluate existing research that has focused on the long-term development of training characteristics and performance-determining factors in male and female endurance athletes reaching an elite/international or world-class level, (2) summarize the available evidence, and (3) point out existing knowledge gaps and provide methodological guidelines for future research in this field.

## Methods

This scoping review was conducted following the Joanna Briggs Institute methodology for scoping reviews [24]. The review protocol and search results for each step of the review are available on the Open Science Framework (https://osf.io/b3fwu/). The Preferred Reporting Items for Systematic reviews and Meta-Analyses extension for Scoping Reviews Checklist (PRISMA-ScR) was followed step by step [25].

An initial limited search of PubMed was undertaken to identify potentially relevant articles. The words contained in the titles and abstracts of relevant articles, and the index terms used to describe the articles, were then utilized to develop a full search strategy. Broad inclusion criteria were initially employed to increase the probability of mapping the existing literature of interest and obtaining a comprehensive list of articles. The search strategy (Table 1), including all identified keywords and index terms, was adapted for use across four major databases: PubMed, PsychINFO, SPORTDiscus, and Web of Science. Boolean search terms were used to link nested concepts.

**Table 1:** Search strategy, including all identified keywords and index terms

MeSH terms	Concept 1	Concept 2	Concept 3	Concept 4
	Athletic level	Population	Sex differences	Training characteristics
Athletes Sports	Elite Professional Medalist Olympic “High performance” “World class” “World champion” “Highly trained”	Endurance Aerobic Cycli* Skier* “Cross country” Skiing Runn* Triat* Biath* Swim* Rowing Rower Orienteer* “Long distance” Marathon Athletics Skating Biking	Female Woman/Women Girl Male Man/Men Boy Sex Gender	Training Endurance Load TRIMP Intensity Speed Velocity Frequency Volume Distance Distribution Time RPE Mode Modality Movement Activity Terrain Periodi?ation Tapering Peaking Altitude Progression Longitudinal

*MeSH* Medical Subject Headings, *RPE* ratings of perceived exhaustion

An asterisk (*) indicates a Boolean operator for truncation searching from the word stem, while a question mark (?) represents a wild card replacement of a single letter

Once the search strategy was completed, search results were collated and exported to EndNote referencing software (version X9.3.3; Clarivate Analytics, Philadelphia, PA, USA). Duplicates were removed using the duplication detection tool of the Endnote software, before all remaining unique records were made available to reviewers for further processing (i.e., study screening and selection). In addition to the systematic search of the four primary databases, an additional search was performed using Google Scholar, with the first 200 results exported for further screening. The initial database search output can be viewed at https://osf.io/b3fwu/.

The types of publications included in the first stage of the literature review were: peer-reviewed journal papers (published between the period 1 January, 1990 and 8 December, 2022, written in English and involving human participants), reviews, and meta-analyses; while non-peer reviewed articles published in magazines, unpublished doctoral dissertations, and masters’ theses were excluded. Both quantitative, qualitative, and mixed-method studies were included to consider different aspects of the development process. To chart data related to long-term development, the studies were included if training or physiological characteristics were reported for ≥ 2 years. The participant classification framework of McKay et al. [26] was used and only studies with participants classified as Tier 4 (elite/international level) or Tier 5 (world-class level) were included.

**Fig. 1:**
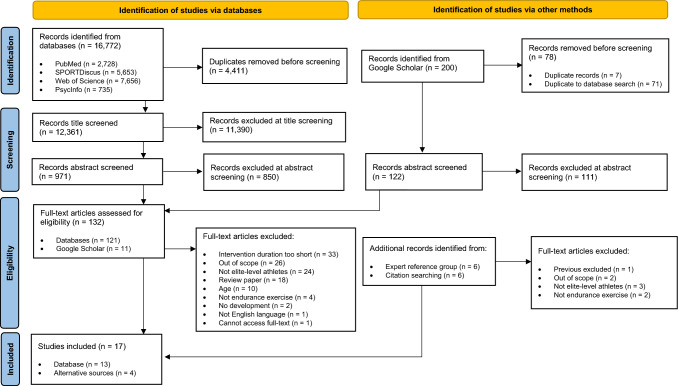
Preferred Reporting Items for Systematic reviews and Meta-Analyses (PRISMA) flow diagram showing the flow of information through the review process [28]. From Page MJ, McKenzie JE, Bossuyt PM, Boutron I, Hoffmann TC, Mulrow CD, et al. The PRISMA 2020 statement: an updated guideline for reporting systematic reviews. BMJ 2021;372:n71. https://doi.org/10.1136/bmj.n71

The review process consisted of three levels of screening: (1) an initial title screening; (2) an abstract review; and (3) a full-text review. Two investigators (HS and JOO) independently screened all articles against the forementioned inclusion and exclusion criteria and then compared results. Where consensus was not reached, it was resolved by means of consolidation with a third independent researcher (GSS). Reasons for the exclusion of any full-text source are reported in the scoping review report. The search results are presented in a PRISMA flow diagram (Fig. 1) [27, 28]. Following the final full-text review screening step, an expert panel (*n* = 6) of experienced academics in exercise physiology and athlete development was assembled to review the included studies and suggest any additional relevant articles that could be considered for inclusion. Snowball searching was also employed on the reference lists of the included studies, to identify any other relevant sources.

A data extraction form was developed and key information on the selected articles, population, concept, and context was collected. This form was reviewed and tested by all research team members before implementation, to ensure that the form accurately captured the necessary data. Extracted study variables included: primary author, year of publication, athletes’ country, study aim/purpose, sample description and size, participant details, study methodology, body composition, training characteristics (TV, TID), physiological characteristics (*V*O_2max_, submaximal responses, performance indicators), and performance. The charting process was an iterative process with three researchers (HS, JOO, and GSS) extracting the data.

## Results

### Study Characteristics

A total of 17 peer-reviewed journal articles were included. Sixteen of these studies used a retrospective study design, with a mean duration of ~ 7 years (range 2–17 years). Out of the 17 studies, ten included men exclusively, five included only women, and two included a mix of men and women. Cumulatively, the studies included a total of 109 participants, with approximately a quarter (*n* = 29; 27%) being women. The two studies that included both sexes represented two-thirds (*n* = 73; 67%) of the total participants, with a total of 24 women and 49 men. The five women-only studies were all individual case studies, accounting for just 5% of the 109 total participants, while men-only studies represented 28% (*n* = 31). A total of 11 different studies were individual case reports. Athletes from seven Olympic endurance sports were represented in the study, middle- and long-distance running (*n* = 41); swimming (*n* = 41); cycling (*n* = 13); rowing (*n* = 6); triathlon (*n* = 6); biathlon (*n* = 1); and cross-country skiing (*n* = 1), while only one athlete represented the Paralympic disciplines (swimming). The majority of included studies (*n* = 11; 65%) were published after 2010. Athletes from seven countries were included, with the majority of athletes (85%) from Spain (*n* = 52) and Australia (*n* = 40), and the remaining from Norway (*n* = 7), Croatia (*n* = 4), UK (*n* = 3), France (*n* = 2), and Belgium (*n* = 1).

### Training Characteristics

The ten studies that provided information about the long-term development of training characteristics are presented in Table 2. Nine of the studies were individual case studies that were conducted on athletes in cross-country skiing, biathlon, running, cycling, rowing, and para-swimming. Six studies described training data that ranged from 6 to 17 years duration. No information about training before a junior age was reported by any of the studies. Table 2 includes a summary of TV and TID from the included studies. Other important training characteristics such as training frequency, strength, speed, and altitude training were rarely described and are not included in Table 2. Specifically, four studies included information about training frequency [29–32], three studies reported strength and speed training [30, 33, 34], and four studies included information about the use of altitude training.[30, 33–35]. One study had a detailed description of the altitude training during the 5 most successful years (30–35 years of age) but no information about altitude training from earlier years was presented [30]. The other studies only briefly described that altitude training was employed, without providing any detailed data.

**Table 2:** Overview of the development of training characteristics and physiological-determining factors

References	Participants Nation and sport *n* (sex) Age (y)	Time period (y)	Training characteristics TV development TID development	Performance-determining factors *V*O_2max_ Submaximal responses Maximal performance indicators
Female population				
[30]	Norwegian XC skier; Tier 5; (♀ = 1); 18–35 y	17	**Age 20**–**35 y.:** ↑TV 522–940 h·y^−1^ (+ 80%) ↑LIT ~ 430 to ~ 800 h·y^−1^ **Age 20**–**23 and 29**–**35 y:** MIT + HIT ~ 60 h·y^−1^ **Age 23**–**28 y:** MIT + HIT ~ 80 h·y^−1^ **Age 20**–**27 y:** LIT/MIT/HIT ~ 88/2/10% **Age 28**–**35 y:** LIT/MIT/HIT ~ 92/3/5%	**Age 30**–**35 y (five most successful seasons):** ↔ *V*O_2max_ = 67.7 ± 1.7 mL·kg^−1^·min^−1^ ↔ vAT = 10.7 ± 0.4 km·h^−1^ (running @10.5% inclination) ↔ AT = ~ 89% of *V*O_2max_
[35]	British marathon runner; Tier 5; (♀ = 1); 19–30 y	12	**Age 18**–**29 y:** ↑TV from 25–30 miles·wk^−1^ to 120–160 miles·wk^−1^ (380–433%) ↑TV from 40–48 km·wk^−1^ to 193–258 km·wk^−1^ (382–438%)	**Age 18**–**29 y:** *V*O_2max_ = ~ 70 mL·kg^−1^·min^−1^ (range 65–80 mL·kg^−1^·min^−1^) ↑v*V*O_2max_ = 20.5–23.5 km·h^−1^ (+ 15%) ↓*V*O_2_ @16 km·h^−1^ = 205–175 mL·kg^−1^·km^−1^ (− 15%) ↑LT = ~ 15.5 km·h^−1^ to ~ 17.5–18.5 km·h^−1^ (+ 13–19%)
[44]	British marathon runner; Tier 5; (♀ = 1); 18–22 y	5		**Age 18**–**22 y:** ↓*V*O_2max_ = 72.8–66.7 mL·kg^−1^·min^−1^ (− 8%) ↑LT = 15–18 km·h^−1^ (+ 20%) ↑v*V*O_2max_ = 19.5–22 km·h^−1^ (+ 13%) ↓VO_2_@16 km·h^−1^ = 53–48 mL·kg^−1^·min^−1^ (+ 9%)
[34]	Norwegian Paralympic swimmer; Tier 5; (♀ = 1); 23–26 y	4	**Age 23**–**26 y:** ↑TV 388–656 h·y^−1^ (+ 69%), 1126–1993 km·y^−1^ (+ 77%) ↔ LIT/MIT/HIT ˃90/2–4/3–6% (of total training)	
[31]	Norwegian marathon runner; Tier 5; (♀ = 1); 25–26 and 29–30 y	2	**Age 25**–**26 y (track focus):** TV = 123 km·wk^−1^ (119–132 km·week^−1^) **Age 29**–**30 y (marathon focus):** TV = 121 km·wk^−1^ (first 26 weeks of the year) ↓TV than current marathon runners Similar TV when changing from track races to marathon races	
Male population				
[29]	Belgium rower; Tier 5; (♂ = 1); 16–30 y	15	**From junior (18 y) to senior (23 y):** ↑TV 4372–6091 km·y^−1^ (+ 39%), 11.3–17.2 h·wk^−1^ (+ 52%) ↑LIT = 4021–5664 km·y^−1^ (+ 40%) ↓MIT = 218–121 km·y^−1^ (-44%) ↑HIT = 87–280 km·y^−1^ (+ 221%)	**Age 16**–**20 y:** ↑VO_2max_ = 4.1–5.3 L·min^−1^ (+ 29%) **Age 20**–**27 y:** ↑VO_2max_ = 5.3–6.0 L·min^−1^ (+ 13%) **Age 27**–**30 y:** ↔ VO_2max_ = 6.0 L·min^−1^ **Age 16**–**27 y:** ↑PO_La4_ = 200–404 W (+ 101%) **Age 27**–**30 y:** ↔ PO_La4_ = 396 W **Age 16**–**25 y:** ↑PO_max_ = 330–536 W (+ 62%) **Age 25**–**30 y:** ↔ PO_max_ = 536 W
[33]	French biathlete; Tier 5; (♂ = 1); 19–31 y	11	**Age 19**–**31 y:** ↑TV = ~ 530–700 h·y^−1^ (+ 32%) **Age 19**–**30 y:** ↔ LIT/MIT/HIT ~ 86/3/4% (of total training) **Age 30**–**31 y:** ↑MIT = 7.4% (of total training)	
[39]	French rower; Tier 5; (♂ = 1); 26–36 y	10		**Age 26**–**32 y:** ↑*V*O_2max_ = 67.6–70.7 mL·kg^−1^·min^−1^ (+ 5%) ↑PO_max_ = 455–461 W (+ 1%)
[40]	Croatian rowers; Tier 5; (♂ = 4); 16–21 y	6		**Age 16**–**20 y:** ↑*V*O_2max_ = 61.5–69.7 ml·kg^−1^·min^−1^ (+ 26%) **Age 20**–**21 y:** ↔ *V*O_2max_ = 69.7 mL·kg^−1^·min^−1^ **Age 16**–**21 y:** ↑PO_AT_ = 297–359 W (+ 21%) ↑PO_VO2max_ = 400–481 W (+ 20%)
[36]	Spanish cyclist; Tier 4; (♂ = 1); 18–23 y	6	**Age 18**–**23 y:** ↑TV = 526–943 h·y^−1^ (+ 79.2%), 14.733–29.383 km·y^−1^ (+ 100%) Large increase (60–62%) before becoming professional, but smaller increases afterwards	
[32]	Norwegian MD runners; Tier 5; (♂ = 3; HI, FI, and JI); 17–28 y	6	**HI age 17**–**21 y:** ↑TV = 100–110 to 156 km·wk^−1^ (~ + 50%) **FI age 17**–**20 y:** ↑TV = 70–80 to 120–130 km·wk^−1^ (~ + 66%) **JI age 17**–**18 y:** TV = 130–140 km·wk^−1^ All had a similar TV = 150–160 km·wk^−1^ in the 2019 preparation period (HI 28 y, FI 26 y, JI 19 y)	
[37]	Norwegian MD runner; Tier 5; (♂ = 1); 17–21 y	5	**Age 17**–**21 y:** ↑TV = 100–110 to 145–160 km·wk^−1^ (~ + 50%) ↑LIT = ~ 80 to ~ 110 km·wk^−1^ (~ + 37%) ↑MIT = ~ 10 to ~ 20 km·wk^−1^ (~ + 100%) ↑HIT = ~ 2 to ~ 3 km·wk^−1^ (~ + 50%) Training recorded 10 weeks. January-March	
[43]	Spanish cyclists; Tier 5; (♂ = 12); 22–27 y	5		**Age 22**–**27 y:** ↔ *V*O_2max_ = range 75.5–77.3 mL·kg^−1^·min^−1^ ↑DE = 24–27%
[42]	Spanish triathletes; Tier 4; (♂ = 6); 24–25 y	2		**Age 24**–**25 y:** ↔ *V*O_2max_ = range 77.8–77.4 mL·kg^−1^·min^−1^ ↔ PO_max_ = range 5.7–5.9 W·kg^−1^) 3-km TT (as running and running after cycling) did not change
[38]	British MD runner; Tier 5; (♂ = 1); 26–27 y	2	**Age 26**–**27 y:** ↔ TV = range 112–114 km·wk^−1^ ↑LIT, ↓MIT, ↑HIT (absolute numbers not reported)	**Age 26**–**27 y:** ↑ *V*O_2max_ 70.5–78.,5 mL·kg^−1^·min^−1^ (+ 11%) ↑LT = 16–18 km·h^−1^ (+ 13%) ↑v*V*O_2max_ = 10.4–23.1 km·h^−1^ (+ 13%)
Mixed population				
[45]	Australian swimmers; Tier 4; (♀ = 16, ♂ = 24); 19–25 y (♂); 18–24 y (♀)	6		**Age 19**–**25 y (♂) and 18**–**24 (♀) y:** ↑LT = 1.2% annual increase (♀) ↑maximal speed = 0.6–1.0% annual increase (♂/♀)
[41]	Spanish MD and LD runners; Tier 4; (♀ = 8, ♂ = 25); 23–26 y (♂); 26–29 y (♀)	3		**Age 23**–**26 (♂) and 26**–**29 (♀) y:** ↔ *V*O_2max_ = ♂ ~ 76 mL·kg^−1^·min^−1^ and ♀ ~ 70 mL·kg^−1^·min^−1^ Running performance (800-marathon): + 1.8% (♂) and + 0.7% (♀)

*AT* anaerobic threshold, *DE* delta efficiency, *HIT* high-intensity training, *LD* long-distance, *LIT* low-intensity training, *LT* lactate threshold, *MD* middle-distance, *MIT* moderate-intensity training, *PO* power output, *TID* training intensity distribution, *TT* time trial, *TV* training volume, *v* velocity, *VO*_*2max*_ maximal oxygen uptake, *XC* cross-country, *y* years, ↑ increase, ↓ decrease, ↔ stabilized, ♀ female, ♂ male

#### Training Volume

In total, eight studies reported a progressive non-linear increase in TV [29, 30, 32–37]. Two female world-class athletes, from cross-country skiing and marathon running, had relatively low TV at a junior age, and increased their TV by 80–500% over a 10- to 12-year period, from 18 to 20 years of age until the age of peak performance [30, 35]. A similar pattern was seen in two male athletes, from rowing and cycling, with a 50–80% increase in TV from the age of 18–23 years [29, 36], in one female para-swimmer with an almost 70% increase in TV from the age of 23–26 years [34] and in two male middle-distance runners from the age of 17–21 years with TV increases of approximately 50% and 66% [32]. In contrast, a much lower increase in TV (30%) was reported in a world-class male biathlete from the age of 21–31 years [33]. Three studies reported a plateau in TV (500–900 h·year^−1^) between the ages of 26–30 years [30, 31, 33]. Particularly large increases in TV were observed to occur relatively early in the development process, such as a 60% increase in TV from the age of 20–24 years in a world-class female cross-country skier and a 60% TV increase from the age of 18–20 years for a male Spanish cyclist [30, 36].

#### Training Intensity Distribution

Training intensity distribution was described in six individual case studies [29, 30, 33, 34, 37, 38]. One of these studies reported increased LIT and MIT, and an associated decrease in the amount of HIT, at a later stage in the career of a female world-class cross-country skier [30]. Two studies showed a change towards a higher volume of both LIT and HIT, but reduced volume in MIT, for male rowers (number of kilometers rowed per week) and long-distance runners (relative distribution) [29, 38]. In contrast, a middle-distance runner reported an increase in the number of kilometers run per week at both LIT, MIT, and HIT from the age of 17–22 years [37]. Finally, a relatively stable TID was reported over 10 years in a world-class male biathlete and over 4 years in a world-class para-swimmer [33, 34].

### Performance-Determining Factors

The 11 studies that describe the development of physiological parameters are presented in Table 2. Five of the studies were individual case studies and described world-class athletes in cross-country skiing, rowing, and running. Only two studies included both male and female athletes.

An increase in *V*O_2max_ was reported in four studies [29, 38–40]. The relative (i.e., body mass normalized) *V*O_2max_ of a male rower increased by 4% from the age of 25 years until he retired at 32 years [39]. Two other studies on male rowers found a 29% absolute to 26% relative increase in *V*O_2max_ from 16 to 20 years [29, 40]. In one of these studies, a further 13% increase was observed from the age of 20–27 years, before stabilizing at 28 years [29]. An 11% increase in relative *V*O_2max_ was also reported in a male middle-distance runner who altered his TID by increasing the proportion of LIT and HIT, but decreasing MIT, over two consecutive seasons [38].

Five studies found no change in relative values of *V*O_2max_ of elite/international and world-class level athletes in long-distance running, triathlon, cycling, and cross-country skiing [30, 35, 41–43]. Six studies described improvements in submaximal performance-determining variables (e.g., lactate/anaerobic threshold and/or economy/efficiency) [29, 30, 35, 38, 40, 44] and six studies showed improvements in performance indicators (e.g., maximal speed, maximal power output, and speed at *V*O_2max_) [29, 35, 38–40, 44] over durations of 2–17 years in world-class runners, cross-country skiers, and rowers.

## Discussion

This scoping review aimed to (1) identify and evaluate existing research that has focused on the long-term development of training characteristics and performance-determining factors in male and female endurance athletes reaching an elite/international or world-class level, (2) summarize the available evidence, and (3) point out existing knowledge gaps and provide methodological guidelines for future research in this field.

In total, 17 studies were included in the review, with all but one using a retrospective study design and the majority of participants being male. A non-linear year-to-year increase in TV was reported for most athletes, resulting in a plateau at the elite/international and world-class levels. Only six case studies reported details about the development of TID, with all showing an increased volume of LIT while the long-term changes in MIT and HIT distribution varied across studies. Improvements in submaximal performance-determining factors (e.g., lactate/anaerobic threshold and work economy/efficiency) and various performance indices (e.g., peak speed/watt during performance testing) were reported for seven of the studies, with inconsistent findings in the ten studies reporting long-term development of *V*O_2max_. No evidence regarding possible sex differences in the development of training or performance-determining variables among endurance athletes reaching an elite/international or world-class level was described for any of the included studies.

### Study Characteristics

Only studies with elite/international or world-class level athletes (i.e., performance level Tier 4 and 5) as classified according to the definition by McKay et al. [26] were included in the review. Accordingly, this criterion decreased the pool of potentially relevant research, and of the included studies, the majority had small sample sizes (*n* < 5). A possible explanation for the limited number of relevant studies is the lack of systematic monitoring of elite/world-class athletes and/or restrictions on publishing unique data from such individuals. It is understandable that athletes may wish minimal distractions during their sporting careers, and that national federations likely want to maintain secrecy to gain a competitive advantage in the short-term perspective. However, we believe that systematic monitoring and publishing of long-term athletic data would benefit the sporting community at large, by contributing to the body of literature regarding elite-level training and athletic development.

The majority of the included research in this scoping review were case studies, which are considered the weakest form of scientific evidence and limit the possibility for generalization of the findings. Still, the case studies provide rich in-depth material on unique world-class level athletes such as Grete Waitz, Paula Radcliffe, Marit Bjørgen, Martin Fourcade, Henrik, Filip, and Jacob Ingebrigtsen, Tim Maeyens, Sarah Louise Rung, and Mo Farah. While studies including more athletes would improve the ability to generalize findings, another possibility would be merging data from several individual case studies of world-class athletes, to produce stronger evidence. However, such assimilation would require a common framework for the reporting of high-quality long-term training data in elite athletes. Overall, implementation of such a policy would require collaboration between sports federations and research institutions, resulting in national and international projects with a concurrent focus on helping today’s athletes optimize their abilities, while the long-term data would enhance the performance of future generations of athletes. Furthermore, the finding that no information about training before a junior age was reported by any of the studies in this review demonstrates the importance of systematically monitoring athletes from a younger age.

Over the past decade, there has been a burgeoning awareness and discussion regarding the lack of female-specific sports science research [46]. The present systematic scoping review highlights that female participants are considerably under-represented, and these findings align with other recent studies that emphasize the continued paucity of research on women in sport [47]. Out of the 17 studies included in this review, only 5% of the participants were from female-only studies. Similarly, Cowley et al. [47] reported that only 6% of randomly sampled sport and exercise studies, published between 2014 and 2020, were on women. Furthermore, the data in this review showed an under-representation of female participants and Paralympic athletes, a small number of unique sports, and a clear predominance of athletes from Western Europe. This restricts the generalizability of the existing scientific evidence and limits the possibility to inform sport practices and policies [48].

### Training Characteristics

Although scientific evidence is lacking, long-term dedicated training is crucial for reaching a world-class level in endurance sports. In our results, only seven of the studies included detailed information about the long-term progression in training; from a junior age or beginning of a senior age (18–20 years), until reaching elite/international or world-class level (i.e., 23–29 years). Interestingly, none of these studies included information about training before the age of 18 years, which could be a topic to investigate in future studies.

The studies demonstrate a non-linear increase in TV, varying from 30 to 500% over periods that range from 2 to 17 years. Such large overall increases in TV required a considerable elevation in TV for specific years. However, more information is needed to understand the observed increases in TV, and if larger increases are associated with a more rapid performance and physiological development, or conversely, a greater risk of stagnation.

Three studies documented a plateau in TV occurring close to peak performance, from ~ 650 to 900 h·yr^−1^ depending on the type of endurance sport and individual needs. This is not unexpected for the long-term development process, as a TV plateau is often observed around the same time an athlete reaches their peak performance level. However, we observed a decrease in TV, although performance level was maintained in the final years of a world-class female cross-country skier [30]. The findings of a gradual TV increase prior to reaching a plateau support the guidelines provided by sporting bodies, but additional research on how training progression can be further optimized is required.

The effectiveness of utilizing TID concepts to maximize endurance adaptations and performance is a “hot topic” in the scientific literature [19, 20, 49]. However, little research has investigated the long-term development of TID in elite/international or world-class endurance athletes. In this scoping review, six case studies detailed athletic TID development, with all studies reporting an increased LIT volume. Two of the studies showed a stable portion of MIT and HIT over time [33, 34]; one study observed a change towards a higher volume for both MIT and HIT [37], while another study showed a small relative increase in MIT and a corresponding decrease in HIT [30]. The remaining two studies described a reduction in MIT and increased HIT [29, 38]. It is therefore difficult to draw any conclusions from this summary. In addition, six studies used different methodologies to determine TID, included athletes from different sports, and detailed different timespans. For example, one study compared only 2 years of training [38], while another study described training changes over 12 years [30]. In addition, the different methodology for logging intensity zones [21] and the complexity of the long-term development process, make it challenging to form generalizations about TID. However, increased LIT was associated with progression in the training load for all studies, and as such, this factor appears to be a critical cornerstone of any successful endurance training program. Accordingly, the proportion or volume of MIT and HIT is a crux of the training debate that has been previously described [20]. Still, an optimal endurance training program should provide the necessary total TV, whilst balancing the appropriate proportion of MIT and HIT for each individual athlete. The current scientific understanding of how TID should be divided over a long duration is limited and more information regarding the long-term development of TID during different stages of an athlete’s career is needed.

### Performance-Determining Factors

The description of a world-class athlete implies a positive performance development across multiple years, and seven of the included studies also reported positive developments of performance and/or maximal performance indicators [29, 35, 38, 40, 44, 45, 50]. However, a compilation of the results is challenging because of testing in different periods of the season, and the fact that these performance determinants appear particularly sensitive to seasonal variations in training.

While high *V*O_2max_ values have been measured in world-class athletes for most endurance sports [51], less data are available on the long-term development of *V*O_2max_. In this scoping review, *V*O_2max_ was reported in ten studies [29, 30, 35, 38–44], and suggests a considerable individual variation in the development of *V*O_2max_ of elite athletes during their athletic careers. These cumulative data indicate that for some athletes, *V*O_2max_ may develop and become optimized in the early stages of their career, while other performance-determining factors then drive subsequent improvements. In contrast, other athletes were able to further develop their *V*O_2max_ at later stages in their careers. The causative reason behind this divergent response may be due to training pattern changes that stress complementary *V*O_2max_-limiting factors during this period. However, this theory should be considered speculative and additional research is required to further investigate this concept. For example, changes in body mass or body composition could change the relative *V*O_2max_ values.

While *V*O_2max_ showed different development patterns in world-class athletes, performance-determining factors that were based on submaximal responses demonstrated considerably more consistent developments, both with and without improvements in *V*O_2max_ [29, 30, 35, 38, 40, 44, 50]. This result provides further support for the concept that endurance performance improvements after the age of 18–20 years are primarily related to other factors than VO_2max_, such as improved fractional utilization of *V*O_2max_ and work economy/efficiency. This is exemplified in the studies of Paula Radcliff [35, 44] who already reached a high value of *V*O_2max_ at the age of 18 years, while improvements in running economy and running performance continued to develop gradually over years.

### Existing Knowledge Gaps

The low number of peer-reviewed articles that have presented data on the long-term development of athletes reaching elite/international or world-class level, in combination with varying data quality and lack of important details, highlights the urgent need for more long-term studies to support evidence-based talent development in sport. As more than half of the included studies were case studies, and most of the data were collected retrospectively, prospective studies would be of particularly interest. The low number of studies in women also confirms their current under-representation in the scientific literature.

Although participation and professionalization in Paralympic sports are increasing, it is problematic that only one study with Paralympic athletes met the inclusion criteria in this systematic scoping review. The same applies for the small number of unique sports and the clear predominance of athletes from Western Europe, which highlights the need for further examinations of different sports, cultures, and ethnicities.

Finally, only four of the 17 studies reported concurrent data of training and performance-determining variables, limiting the ability to identify potential associations between relevant variables of interest. In this context, future long-term development studies should follow a common framework, enabling the possibility to compare data across studies and the performance of future meta-analyses.

### Methodological Guidelines for Future Research

The findings in this scoping review demonstrate that a common methodological framework to permit a detailed comparison between different studies is needed. Based on the findings in this study, we have devised the following guidelines regarding the type of information to include, and the standardization required, for all future studies that wish to report on long-term training development and performance-determining factors in endurance sports (see Table 3). We hope that these guidelines can assist future studies to standardize the collection and presentation of training data, and we encourage other researchers to further develop and validate this proposed framework.

**Table 3:** Methodological guidelines for future research focusing on the long-term development of endurance athletes

Topics	Information and standardization
Time frame	Years without using training diary/logs: qualitatively describe the training/activity background until the start of systematically logging Years with the use of training diary/logs: record daily/weekly training from the year they started logging training until the end of their career
Performance development	Logging of all competitions (type, duration) Logging of results from major events (national and international championship and World Cup as junior and senior)
Training characteristics	Training volume Training frequency Training form (endurance, strength, and speed) Exercise mode (modality) TID 3-zone model (LIT, MIT, HIT) Session design (continuous or interval and choice of terrain) Mobility Qualitative descriptions of methodology used to record TID, TV, and pauses between intermitted training methods (interval training)
Recovery parameters	Rest days Sleeping time and quality Nutrition Qualitative registrations of other loading factors: Work/school or other cognitive stress Traveling (including time-zone changes) Environmental (heat, cold, humidity, altitude) Traumatic challenging emotional events/situations
Health parameters	Illness and injury days Menstrual or hormonal contraceptive cycle
Periodization phases	Annual General preparation Specific preparation Competition period Altitude Tapering
Anthropometric and physiological parameters	Body height (cm) Body mass (kg) Lean body mass (kg) Total body fat (%) Systematic measurements of *V*O_2max_ (L·min^−1^, mL·kg^−1^·min^−1^) speed_@VO2max_, HR_@VO2max_, VE_@VO2max_ Performance indices (peak/average speed and power) Peak/max HR Threshold concepts (LT, VT, CP) speed_@AT_, watt_@AT_, HR_@AT_, lactat_@AT_, VO_@AT,_ PO_@AT_ Work economy or efficiency Relevant speed and strength measurements if possible

*AT* anaerobic threshold, *CP* critical power, *HIT* high-intensity training, *HR* heart rate, *LIT* low-intensity training, *LT* lactate threshold, *max* maximum, *MIT* moderate-intensity training, *PO* power output, *TID* training intensity distribution, *TV* training volume, *VE* minute ventilation, *VO* oxygen uptake, *VO*_*2max*_ maximal oxygen uptake, *VT* ventilatory threshold

## Conclusions

The current review found that only a handful of previous studies have reported the long-term development of training characteristics and performance-determining factors in male and female endurance athletes reaching an elite/international or world-class level. There are particularly limited data on women, and athletes aged younger than 18 years. No evidence was found for possible sex differences. Although 17 studies were included in this systematic scoping review, athletes from only a small number of countries and sports are described. Accordingly, current long-term talent development practices in endurance sports have insufficient scientific evidence.

The training characteristics described a non-linear year-to-year increase in TV for most world-class endurance athletes, subsequently resulting in a plateau. However, the progression of TID showed individual patterns. While it is likely that a gradual progression in TV, with most of the increase stemming from more LIT, is required to reach a high level in endurance sports, no pattern was identified for the optimal development of MIT and HIT. The few studies reporting the development of performance-determining variables indicated a consistent improvement in maximal performance tests and submaximal performance indicators for most athletes. Conversely, *V*O_2max_ development was observed to be inconsistent.

Overall, there is an urgent need for additional research that describes the long-term development of world-class athletes. Specifically, the implementation of systematic monitoring of athletes from a young age, employing high-precision reproducible measurements of training and performance-determining variables would enable prospective and high-quality retrospective study designs of considerable scientific and practical value. In addition, the use of a common methodological framework is also necessary to permit a detailed comparison between different studies and allow for future meta-analyses.
